# Interplay between Herpesvirus Infection and Host Defense by PML Nuclear Bodies

**DOI:** 10.3390/v1031240

**Published:** 2009-12-15

**Authors:** Nina Tavalai, Thomas Stamminger

**Affiliations:** Institute for Clinical and Molecular Virology, University of Erlangen-Nuremberg, Schlossgarten 4, 91054 Erlangen, Germany; E-Mail: nina.tavalai@viro.med.uni-erlangen.de

**Keywords:** herpesvirus, nuclear domain 10, PML nuclear bodies, PML, Sp100, hDaxx, antiviral defense, intrinsic immunity, interferon

## Abstract

In recent studies we and others have identified the cellular proteins PML, hDaxx, and Sp100, which form a subnuclear structure known as nuclear domain 10 (ND10) or PML nuclear bodies (PML-NBs), as host restriction factors that counteract herpesviral infections by inhibiting viral replication at different stages. The antiviral function of ND10, however, is antagonized by viral regulatory proteins (e.g., ICP0 of herpes simplex virus; IE1 of human cytomegalovirus) which induce either a modification or disruption of ND10. This review will summarize the current knowledge on how viral replication is inhibited by ND10 proteins. Furthermore, herpesviral strategies to defeat this host defense mechanism are discussed.

## 1. Introduction

In general, viruses depend in many aspects on the cellular machinery in order to replicate efficiently. Consequently, viruses have evolved in tight association to the host cell to be able to hijack the cellular apparatus that is necessary for their replication. During co-evolution, however, the cell has developed various antiviral defense mechanisms in order to limit viral replication and spread, which viruses have to cope with. In particular, it could be shown that a set of constitutively expressed cellular proteins, collectively termed intrinsic immunity, represents one of the first lines of antiviral defense in naive hosts [1]. Approximately 15 years ago a subnuclear structure known as nuclear domain 10 (ND10; alternatively termed PML-NBs for PML nuclear bodies or PODs for PML oncogenic domains) has been discovered that is targeted by a variety of viruses belonging to different viral families. Although there is now increasing evidence that ND10 proteins mediate intrinsic immunity against viruses, there has always been a controversial debate about the functional significance of ND10 for viral replication. The main objective of this article is to critically discuss the very recent literature on the role of ND10 for viral infection. It will focus on herpesviruses, since these have been most extensively studied with respect to the functional consequences of ND10 association. The studies highlighted in this review provide accumulating evidence for an important role of ND10 in host antiviral defenses against herpesviruses. Finally, the diverse strategies herpesviruses have evolved to overcome this ND10-based host defense are outlined.

## 2. Structural Aspects of ND10

Nuclear domains 10 (ND10), also referred to as nuclear dots, PML nuclear bodies (PML-NBs), or PML oncogenic domains (PODs), can be defined as dynamic, spherical, macromolecular structures which represent accumulations of multiple cellular proteins that assemble in distinct foci within the interchromosomal space of the nucleus [2]. Like many transcription or RNA processing factors, ND10 bodies are associated with the nuclear matrix, since treatment with RNase or DNase does not alter their morphology [3,4]. The apparent size of PML-NBs ranges from 0.2 to 1 μm, and their frequency depends on cell type and status, varying from two or three to as many as 30 per cell [4]. Presently, the nuclear protein database lists 70 proteins that have been reported to be present in or associated with ND10 structures (http://npd.hgu.mrc.ac.uk/). These proteins can be subdivided into two classes, based on whether they are constitutively or transiently present at ND10. Permanently localized at ND10 are the major components PML (promyelocytic leukemia protein), hDaxx, Sp100 (speckled protein of 100 kDa), SUMO-1 (small ubiquitin-related modifier 1), and the Bloom syndrome helicase BLM [2]. On the contrary, quite a variety of factors can be found at ND10 only under certain conditions (e.g., components of the DNA repair machinery) [5–7] or upon overexpression (e.g., BRCA1–breast cancer protein 1) [2,8]. However, in the latter case, it has to be questioned, whether such polypeptides constitute natural ND10 components, as more and more data suggest a role of ND10 in the degradation of mis- or unfolded protein aggregates [9,10]. Thus, as the composition of ND10 varies both within and between cells, it is evident that ND10 represent functionally heterogeneous protein accumulations. The Sp100 protein was the first ND10 constituent identified using sera of patients suffering from the autoimmune disease primary biliary cirrhosis (PBC) [11]. Thereafter, PML was discovered as the defining factor of ND10. It functions as a kind of scaffold protein that is responsible for the assembly and maintenance of PML-NBs and recruits other ND10-associated proteins like hDaxx to this subnuclear structure [12,13]. Since PML constitutes the key component of ND10 integrity, loss of PML consequently leads to a dispersal of ND10-resident proteins as observed in mouse PML-null fibroblasts [12,13] or human PML-knockdown (PML-kd) fibroblasts [14,15].

## 3. The Major ND10 Constituents and Their Functions

### 3.1. PML

The PML protein was originally discovered in patients suffering from acute promyelocytic leukemia (APL), where a reciprocal chromosomal translocation resulting in a fusion of the PML protein to the retinoic acid receptor α turned out to be responsible for this hematopoietic malignancy [16]. PML, also known as TRIM19, belongs to the RBCC or tri-partite motif family (TRIM) of proteins that are comprised of a zinc-finger RING domain, one or two B-boxes (cysteine/histidine-rich motifs) and a predicted α-helical coiled-coil domain [17]. This motif, which allows PML to interact with other proteins as well as to homo-oligomerize, is essential for ND10 formation and for the function of PML as a growth- and transformation-suppressor [18]. Due to differential splicing of the PML gene transcript which consists of nine exons, at least seven different PML isoforms (I to VII) are expressed within cells, all sharing a common N-terminus but varying in their C-termini [17]. Since not all PML isoforms retain the nuclear localization signal in exon 6, beside nuclear ND10-localized PML also cytoplasmic species of PML exist [17]. So far, the functions of the PML splice variants are not fully understood. However, in this context it is of note that recent publications demonstrate an influence of herpesvirus infections on the splicing pattern of PML suggesting diverse roles for the individual PML isoforms with regard to viral replication [19,20]. Besides determining the subnuclear distribution of PML, alternative splicing could add new functional domains to the protein or may be an important mechanism for generating differential PML-binding interfaces for a variety of factors.

Moreover, all isoforms having molecular weights ranging from 48 to 97 kDa (kilo Dalton), are subject to posttranslational modifications like phosphorylation [21] or conjugation to the ubiquitin-homologous protein SUMO (SUMOylation) [22,23]. In addition to three SUMO modification sites at lysine residues 65 (in the RING finger domain), 160 (in the B1 box), and 490 (in the nuclear localization signal) [24], PML also contains a SUMO binding motif that enables it to interact noncovalently with SUMO [25]. Covalent as well as noncovalent SUMO modification is extremely important for the function of PML to orchestrate ND10 formation as clearly illustrated in case of the recruitment of hDaxx to this subnuclear structure [13,25,26].

### 3.2. hDaxx

HDaxx is a highly conserved nuclear protein that contains a serine/proline/threonine-rich domain, an acidic domain, a coiled-coil region, and two paired amphipathic helices [27–30]. It has been recognized as a regulator of both apoptosis and gene expression [31]. In gene regulation, hDaxx has been shown to function as a transcriptional corepressor. It negatively affects gene expression by suppressing the activity of several transcription factors, including Ets-1 (E twenty-six 1) [32], NF-κB (nuclear factor κB) [33], Pax3 [27], E2F-1 [34], Smad4 [35], p53 family members [36], and glucocorticoid, mineralocorticoid and androgen receptors [37]. Recent findings indicate that hDaxx associates via its newly identified SUMO-interacting motif (SIM) with SUMOylated DNA-binding transcription factors [26], thereby recruiting proteins involved in transcriptional repression, such as histone deacetylase 1 (HDAC1) [29], HDAC2 [38], DNA methyltransferase 1 (DNMT1) [39], or ATRX (α-thalassaemia/mental retardation syndrome X-linked) [40,41], a member of the SNF2 family of chromatin remodeling enzymes, to targeted promoters. The transrepressive effect of hDaxx, in turn, is modulated by its subnuclear compartmentalization. The SIM enables hDaxx to also noncovalently interact with SUMOylated PML, resulting in the sequestration of nucleoplasmic hDaxx to ND10 [25,26], which is accompanied by attenuation of its inhibitory function [29]. Beside ND10, hDaxx can also be targeted to the nucleolus, centromeres, or heterochromatin through its interactions with either the nucleolar protein MSP58 (microspherule protein of 58 kDa) [42], the centromeric protein CENP-C [30], or the heterochromatin-associated factor ATRX [40]. Taken together, these observations imply that, depending on its subnuclear localization, hDaxx seems to fulfill distinct functions.

### 3.3. Sp100

Like PML, the Sp100 protein represents a permanent ND10 constituent which is expressed via alternative splicing from a single gene transcript giving rise to four different isoforms designated as Sp100A [11], Sp100B [43], Sp100C [44], and Sp100-HMG [45,46]. All Sp100 splice variants share a common N-terminus harboring an HSR (homogeneously staining region) motif responsible for homo-oligomerization of Sp100 and its targeting to ND10 as well as a binding site for the nonhistone chromosomal DNA-binding protein HP1 (heterochromatin protein 1). The larger splice variants Sp100B, -C, and -HMG encode additional functional motifs at the C-terminus such as a SAND domain (common to all three longer isoforms), a PHD finger-bromodomain (Sp100-C), or an HMG box (Sp100-HMG). All three motifs represent potential DNA-binding domains which can frequently be found in proteins affecting chromatin structure [47]. Together with the observation that Sp100 interacts with heterochromatin protein HP1 [45], which plays a central role in establishing a stable heterochromatic network, this suggests a role of Sp100 in transcriptional regulation. Indeed, the isoform Sp100B has been shown to function as a transcriptional repressor of both cellular and viral promoters in transient expression experiments [48]. In addition, as recently reported, Sp100B preferentially associates via its SAND domain with DNA sequences containing unmethylated CpG dinucleotides, since methylation of cytosines in the CpG context abrogates DNA binding of Sp100B [49]. This finding prompted the authors to speculate that the preference of Sp100B for nonmethylated CpGs could provide a mechanism to specifically target this isoform to foreign DNA (including viral genomes), which is predominantly hypomethylated [49].

Like many ND10 constituents, including PML and hDaxx [23,50,51], Sp100 is also subject to posttranslational modification by SUMO [51]. While SUMOylation of Sp100 is not required for its ND10 localization [52], it has been shown to enhance the interaction with HP1 and thus to stabilize Sp100-HP1 complexes suggesting that the functional interplay of Sp100 between ND10 and heterochromatin could be regulated in this way [44]. Recent data indicate, that the SUMO modification status as well as the relative expression level of Sp100 are to some extent regulated, either directly or indirectly, by PML [14]. SiRNA-mediated knockdown of PML resulted in an apparent reduction in the abundance of Sp100-HMG and the SUMO-modified form of Sp100A, with a concomitant increase in unmodified Sp100A [14]. Thus, Sp100 metabolism seems to be closely linked to that of PML.

## 4. Functions of ND10

Unlike other, more specialized subnuclear structures such as Cajal and Polycomb group bodies, PML-NBs are functionally promiscuous as they represent cellular organizing centers for the coordinated regulation of diverse cellular processes. The research in a variety of fields has produced a substantial literature that links ND10 to oncogenesis [53], DNA damage repair [6], apoptosis [54,55], stress response [56], senescence [57], the ubiquitin pathway [58] as well as to the regulation of gene expression [59]. Despite these various cellular responses, the functions of ND10 are still not fully understood.

In the absence of a clear understanding of the biochemical function of ND10, the following, not mutually exclusive, models have been proposed to explain how this subnuclear structure could exert its biological functions [2,59–62]: (i) PML-NBs might operate as nuclear depots or storage sites for the accumulation of proteins both under pathological conditions to sequester foreign or misfolded proteins as well as under normal conditions to accumulate proteins that can be released or relocated elsewhere as required in order to control their availability at nucleoplasmic sites other than ND10; (ii) PML-NBs may illustrate “catalytic surfaces” where the post-translational modification of proteins takes place (e.g. SUMOylation); (iii) ND10 domains could be active sites for defined nuclear functions such as transcriptional and chromatin regulation.

With respect to the latter activity, evidence continues to accumulate that ND10 play a role for transcriptional regulation since numerous transcription factors and transcriptional regulators dynamically localize to these domains [59] and nascent RNA has been detected in the immediate vicinity of PML-NBs [63]. Nevertheless, the exact molecular mechanisms of ND10-mediated transcriptional regulation remain elusive. Several studies indicate that PML-NBs might regulate transcription by modulating the nucleoplasmic availability or the activity status of transcription factors [59,64]. While not contradicting this model, another emerging idea is that ND10 could also control transcriptional activities indirectly on an epigenetic level by participating in chromatin-remodeling processes. For the ND10 components PML and hDaxx, an association with histone deacetylases (HDACs) [29,38,65] or DNA methyltransferases [66,67], which all exhibit a transcriptionally repressive function, has been demonstrated. Certain PML isoforms, in addition, have been shown to form complexes with multiple corepressors like c-Ski, N-CoR, mSin3A [68], or the novel KRAB-zinc finger repressor PAROT (PML-associated repressor of transcription) [69] as well as to silence transcription by recruiting the histone methyltransferase SUV39H1 [70] or the polycomb group (PcG) protein EZH2 [71], one of the core components of the Polycomb repressive complexes (PRC) 2/3/4. Similarly, Sp100 also behaves as a transcriptional repressor by interacting with the heterochromatin protein HP1 [45,48]. Thus, the fact that several ND10 components associate with potent repressors of gene expression gave rise to the idea of ND10 acting as sites of transcriptional repression. In contrast, however, the presence of transcriptional activators like the acetyltransferase CBP or p53 at ND10 has likewise been described [72,73], reflecting the still controversial debate about the role of this subnuclear structure in regulating gene expression.

Finally, as will be discussed in this review, ND10 have been implicated to play an important role during viral infection [5,62,74–76]. For instance, there are several indications that ND10 are linked to the interferon-mediated antiviral response of the cell [77,78]. Many ND10 proteins, including the major components PML and Sp100, are interferon (IFN) inducible [77,79,80]. Although PML and Sp100 are expressed in the absence of IFN, their expression is greatly increased and directly induced by type I (IFN-α and IFN-β) as well as type II (IFN-γ) interferons through an “IFN-stimulated response element” (ISRE) and an “IFN-gamma activation site” (GAS) which are located within the promoters of their genes [81,82]. In addition, PML transcription can also directly be activated by the IFN-regulatory factor 3 (IRF3), which is also capable of binding to the PML promoter [83]. As a consequence of this, in response to interferon treatment of cells, the number, size, and intensity of PML-NBs increases [84]. The study of ND10 structures in the context of viral infection provides further evidence to implicate ND10 in the IFN pathway. As specified in detail in the sections below, many viruses have evolved polypeptides in order to compromise ND10 integrity to variable extents. Such kind of structural modifications (e.g. dispersal) of ND10 have been shown to frequently correlate with the efficiency of viral infection and thus could be viewed as part of a viral strategy to evade an antiviral function of ND10 [78]. The following chapters will summarize the current state of knowledge concerning the potential role of ND10 during infection with different herpesviruses.

## 5. ND10 during Herpesvirus Infections

Interest in the interaction between viruses and ND10 was first sparked by the observation that infection by herpes simplex virus type 1 (HSV-1) caused a rapid destruction of this subnuclear structure [85]. Subsequently, it was found that the parental genomes and replication complexes of HSV-1 were preferentially located in close association with ND10 [86,87]. This observation has been extended to include members of all subfamilies of the herpesviridae [74,76]. Thus, it appears to be a general feature of nuclear-replicating herpesviruses that their parental genomes preferentially become associated with ND10, and that their initial sites of transcription and the development of DNA replication centers are frequently juxtaposed to these domains or their remnants. The intimate spatial association between herpesviruses and ND10 has raised the important question about the functional consequences of this tight interaction. Some progress has been made within the last years towards answering this question.

## 6. Alpha- Herpesviruses and ND10

### 6.1. Herpes Simplex Virus Type 1 (HSV-1)

HSV-1, a member of the neurotropic alpha sub-family of herpesviruses, is a common human pathogen that causes recurrent infections through its ability to establish a latent state in sensory ganglia after a primary infection of epithelial cells. It was the first virus to be shown to affect ND10 morphology during infection. The initial observation that HSV-1 disrupts ND10 but an ICP0-deficient mutant does not [85], rapidly led to the identification of ICP0 being necessary and sufficient for this effect [88,89]. ICP0 is a RING finger protein that is very important in certain cell types for initiating viral lytic infection and contributes to the reactivation of quiescent viral genomes in cultured cells as well as of latent virus from neurons in mouse models [90]. ICP0 initially precisely colocalizes with ND10 at early times upon infection and subsequently mediates the disaggregation of PML-NBs by inducing the degradation of the SUMO-1 modified forms of PML and Sp100 [91–94], leading to the release and dispersal of other ND10 proteins. This effect of ICP0 on ND10 is dependent upon ICP0 having an intact RING finger motif [88] and reflects its ability as an E3 ubiquitin ligase to recruit the E2 ubiquitin-conjugating enzymes UbcH5a and UbcH6 in order to target specific proteins for degradation by the proteasome [95,96]. However, it is likely that the apparent loss of the low molecular weight isoforms of Sp100 (except Sp100-A) during wt HSV-1 infection is a consequence of ICP0-induced degradation of PML, rather than being a direct effect of ICP0 on Sp100 itself (since the electrophoretic pattern of Sp100 mimics what is observed in PML-depleted cells) [14]. Thus, it is tempting to speculate that by downregulating PML, ICP0 indirectly targets both major ND10 components.

Disruption of ND10 by ICP0 correlates with its role in stimulating HSV-1 lytic infection and reactivation from quiescence or latency [90]. Furthermore, it has been shown that an ICP0-negative HSV-1, which fails to disrupt ND10 is hypersensitive to the effects of IFN in certain cultured cell lines [97,98] and exhibits low pathogenicity in normal mice *in vivo*, while infection of mice that are unable to mount an interferon response restores its replication capacity [99]. Although neither infection with wt nor ICP0-deficient HSV-1 is enhanced in the absence of PML in PML^–/–^ murine embryonic fibroblasts (MEFs) compared to control PML^+/+^ MEFs, the replication of the ICP0-deletion virus is substantially compromised by IFN treatment of these cells in the presence but not absence of PML [100]. These results suggest that PML contributes to the cellular IFN-mediated restriction of HSV-1 infection and that one function of ICP0 is to efficiently counteract the repressive effect of PML. However, neither exogenous expression of PML isoforms III, IV, nor VI turned out to affect virus yield, even though PML overexpression blocked the dispersal of ND10 in response to HSV-1 infection [91,101,102]. In light of the increasing evidence that different PML isoforms possess distinct properties, one possible explanation could be that the PML variants used in those studies may play a minor role in the host IFN-mediated antiviral response. In addition, in contrast to the prior fixed cell analyses, live-cell imaging experiments clearly demonstrated that large ND10 aggregates as a consequence of high-level expression of PML are nonetheless subject to extensive modification in terms of both content and morphology during HSV-1 infection [101].

In principle, it was not until the generation of physiologically more relevant knockdown cells being derived from the natural human host, in which individual ND10 components were depleted, that substantial advances in understanding the biological relevance of ND10 for HSV-1 replication were made. Although infection of cells with an shRNA-mediated down-regulation of either PML or Sp100 had no effect on wt HSV-1 replication, it resulted in a significant increase in the efficacy of gene expression and plaque formation of an ICP0-null mutant virus [14,103]. On the same theme, Negorev *et al.* previously reported that the restrictive activity of Sp100 is based on isoforms B, C, and HMG having an intact SAND domain with which they are capable of repressing viral IE transcription at the promoter level [104,105]. In accordance with this, only overexpression of Sp100B but not Sp100A suppressed HSV-1 gene expression [48,105]. Moreover, by specifically depleting the repressive Sp100 variants B, C, and HMG using RNA interference, these proteins could be confirmed as an essential part of the IFN-mediated restriction of HSV-1 replication [105]. In continuative studies Negorev *et al*. [104]., in addition, demonstrated that IFN treatment of cells leads to a change in the splicing pattern of Sp100 transcripts in favor of the repressive isoform Sp100C

Interestingly, simultaneous knockdown of both ND10 proteins, PML as well as Sp100, further stimulated gene expression and plaque forming ability of the ICP0-knockout virus but did not completely eliminate its replication defect indicating the involvement of additional repressive factors [103]. Indeed, unpublished data presented by the Everett group at the 34^th^ International Herpesvirus Workshop suggest repressive roles for further ND10 constituents such as the transcriptional repressor hDaxx or the chromatin-remodeling enzyme ATRX which cooperatively contribute to the ND10-based inhibition of HSV-1 infection by forming a chromatin-remodeling complex (Everett, R., personal communication). Taken together, these findings add more weight to the concept of ND10 as an intrinsic antiviral defense mechanism of the cell as individual ND10 components participate independently in the silencing of viral gene expression. Furthermore, a recent publication suggests that not only the nuclear ND10-associated PML variants but also the cytoplasmic isoforms could potentially contribute to an antiviral response against HSV-1 as they are capable of sequestering the viral transactivator protein ICP0 to the cytoplasm thereby limiting viral protein accumulation and replication [20]. Interestingly, HSV-1 infection has been shown to induce changes in the splicing of PML pre-mRNA resulting in a selective enrichment of the suppressive cytoplasmic PML variants [20]. In this regard, it is noteworthy that splicing of PML pre-mRNA is also affected by the closely related herpes simplex virus type 2 (HSV-2) [19]. Nojima and coworkers recently identified the viral effector protein ICP27 as an alternative splicing regulator of the PML transcripts which has the ability to actively alter the PML expression profile during HSV-2 infection [19].

### 6.2. Varicella Zoster Virus (VZV)

Like HSV-1, varicella zoster virus (VZV), the causative agent of chicken pox (varicella) in children or shingles (zoster) upon reactivation in elderly or immunocompromised individuals, is characterized as a human alpha-herpesvirus [106]. Comparable to the situation with HSV-1, new findings indicate that VZV infection is also negatively regulated by components of ND10 [107]. The analysis of VZV propagation in cells lacking the major ND10 factors revealed that replication of wt VZV is accelerated in case PML or hDaxx expression is silenced, while depletion of Sp100 had no effect on viral growth [107]. Like all members of the alpha-herpesvirus subfamily, VZV codes for an ortholog of HSV-1 ICP0 termed ORF61p which, similar to ICP0, transcriptionally activates viral promoters and enhances the infectivity of viral DNA [108,109]. Since accumulating evidence suggests that the transactivator function of ICP0, at least in part, is based on the ability of ICP0 to inactivate the cellular intrinsic defense instituted by ND10, a similar role was assumed for ORF61p. However, in contrast to ICP0 which is known to downregulate both restriction factors, PML as well as Sp100, ORF61p was found to specifically reduce Sp100 protein levels only in order to allow efficient virus replication [107,110]. Consequently, a chimeric HSV-1 mutant, which expressed ORF61p in place of ICP0 only partially rescued the phenotype of an ICP0-null virus as ORF61p fails to target PML, which remains available to restrict virus replication [110]. These findings suggest that ICP0 and ORF61p have evolved separately to provide different functions during virus infection. Thus, although alpha-herpesviruses like HSV and VZV are considered to be very similar, the studies presented by the Silverstein group demonstrate that they have evolved unique and specialized ways to interfere with the ND10-mediated host cell response to ensure optimal viral replication.

## 7. Beta-Herpesviruses and ND10

Also in case of HCMV, the prototype of the β-subgroup of herpesviruses, parental viral genomes associate with ND10 followed by the targeting of newly synthesized viral IE transactivators IE1 and IE2, which are essential for initiating the lytic replication program of HCMV [111], to this subnuclear structure [112,113]. The interplay between HCMV nucleoprotein complexes and ND10 structures, however, can only be observed within a narrow time window due to the action of the IE1 protein, which is responsible for the disruption of ND10 during infection with HCMV [112,114,115]. As a mechanism for this, it has been proposed that IE1 abrogates the SUMOylation of PML [116,117], but in contrast to ICP0 of HSV-1 (see 6.1.), IE1 does not require proteasome activity for this effect [118], nor does it possess any intrinsic desumoylation activity *in vitro* [119]. Thus, although the biochemical basis for ND10 disruption by IE1 remains unclear, structural modification of ND10 has been shown to correlate with the functional activities of IE1 in transcriptional regulation, resulting in increased efficacy of viral replication. In the same context, the HCMV infectious cycle, on the contrary, is significantly attenuated in cells expressing high levels of exogenous PML due to a delay in IE1-mediated ND10 dispersal leading to impaired establishment of replication centers and reduced production of early and late proteins [120] (our own unpublished observations). Consequently, these data already implied a repressive function of PML on HCMV replication which is counteracted by the IE1-induced reorganization of ND10. Conclusive evidence for this assumption was finally obtained from infection studies using cells being devoid of genuine ND10: extensive, siRNA-mediated depletion of PML in primary human fibroblasts significantly increased the plaque-forming efficiency of HCMV as a result of an augmented IE gene expression [15]. This effect was considerably enhanced after infection of PML-kd cells with an IE1-deficient HCMV, since loss of PML complemented the growth defect of this mutant virus [15]. Thus, the clear demonstration of an intrinsic antiviral activity of the main ND10 component PML was an important step forward in understanding the functional significance of the intimate relationship between HCMV and ND10.

The interaction of HCMV proteins with ND10 is not exclusive to the IE proteins IE1 and IE2. The viral tegument protein and transactivator pp71 likewise accumulates at ND10 immediately upon HCMV infection and before the production of IE proteins [121–123]. Direct binding to the ND10 component hDaxx has been shown to be responsible for the targeting of pp71 to PML-NBs [121,122]. Interestingly, abolishing pp71′s ability to interact with hDaxx blocks pp71′s localization at ND10 and inhibits pp71′s ability to transactivate the major immediate early promoter (MIEP) of HCMV [121,122,124], indicating that the association of pp71 with hDaxx in ND10 is critical for its function as a facilitator of IE gene expression at the very start of a lytic infection. Although initial studies implied that pp71 and hDaxx associate at ND10 to cooperatively activate the MIEP [121], it is now clear from multiple subsequent work that hDaxx actually silences the MIEP, and that pp71 relieves this repression [125–129]. Consistent with this assumption, overexpression of the cellular restriction factor hDaxx abolishes HCMV infection while downregulation of hDaxx by usage of small interfering RNA (siRNA) technology, on the contrary, results in increased gene expression and virus replication [125–129]. The hDaxx-mediated repression of viral IE gene expression correlates with changes of the chromatin structure around the MIEP as knockdown of hDaxx results in loss of transcriptionally repressive and gain of transcriptionally active chromatin at the MIEP [125]. This regulation appears to involve histone deacetylases, since treatment of infected cells with HDAC-inhibitors relieves the repression of viral IE gene expression [125,126].

In order to successfully antagonize hDaxx-mediated intrinsic immune defense, tegument-delivered pp71 induces the degradation of hDaxx at the very start of a lytic infection [126,129], which has been postulated to occur in a proteasome-dependent [126] and ubiquitin-independent manner [130]. Inhibiting the proteasome at the time of infection, however, still suppresses viral IE gene expression even in the absence of hDaxx, suggesting the existence of additional antiviral targets for proteasomal degradation [131]. Additionally, pp71 has been shown to promote the SUMOylation of hDaxx thereby increasing the basal level of SUMO-conjugated forms of the transcriptional repressor during infection [132]. However, the role this posttranslational modification plays in regulating hDaxx activity remains enigmatic. Interestingly, recent data from the Preston group revealed that prior to the removal of hDaxx, pp71 first stimulates the release of the chromatin-remodeling protein ATRX from ND10 [133]. ATRX has been shown to be sequestered to PML-NBs in an hDaxx-dependent manner where both proteins associate to form a chromatin-remodeling complex. The displacement of ATRX from ND10 contributes to the role of pp71 in alleviating the repression of viral IE gene expression [133].

Consistent with a function of pp71 in counteracting the antiviral effects of hDaxx or ATRX, HCMV inefficiently enters productive infection in the absence of pp71 [124,134], unless hDaxx or ATRX proteins are depleted prior to infection, thus annihilating the impaired growth phenotype associated with a pp71-deficient mutant [127–129]. Failure of pp71 to overcome hDaxx repression blocks viral IE gene expression and may promote the establishment of latent HCMV infections [135]. However, whether hDaxx is a major factor contributing to the control of HCMV latency is still controversially discussed [135,136].

In summary, these data clearly identified the ND10 proteins PML, hDaxx and ATRX as cellular restriction factors responsible for silencing of HCMV IE gene expression directly upon infection. Moreover, since knockdown of hDaxx in combination with PML led to a further increase in the replication efficacy of HCMV [129], this strongly argues for an independent involvement of individual ND10 constituents in the restriction of viral infection and gives rise to the following working model: Immediately after infection, incoming viral genomes are targeted by ND10. It is assumed that ND10 proteins contribute to the formation of a repressive chromatin structure on intranuclear viral DNA via epigenetic processes resulting in transcriptionally inactive viral genomes [137], as many of the ND10 factors have been implicated in epigenetic regulation [138]. In fact, evidence for a “pre-IE” inhibition of viral gene expression instituted by histone posttranslational modifications has recently been provided by Groves *et al*. (2009) [139]. As a first line of defense, the imported HCMV structural protein pp71 antagonizes ATRX-mediated gene silencing by inducing the dissociation of ATRX from ND10. Thereafter, pp71 inactivates hDaxx′s antagonistic function by promoting its proteasomal degradation. The pp71-dependent relieve of ATRX- as well as hDaxx-related repression, then, allows the initiation of viral IE gene expression. The synthesis and accumulation of high levels of IE1 at ND10, in a next step, efficiently overcomes PML-mediated repression by inducing the disruption of this subnuclear domain. This results in modification of the chromatin structure around early and late gene promoters driving a regulated cascade of viral lytic gene expression thereby ensuring an efficient virus production [137,139,140]. In case pp71 is sequestered in the cytoplasm of infected cells, as postulated for cells in which latent-like HCMV infections are established [135], input viral genomes are likewise targeted by ND10. However, the repressive effect of hDaxx on viral gene expression is not relieved as pp71 fails to enter the nucleus and to degrade hDaxx. As a consequence of this, the viral genomes remain in a transcriptionally repressed quiescent state.

Interestingly, recent unpublished observations of our group indicate that, comparable to the situation in PML-, hDaxx- or ATRX-kd cells, ablation of Sp100 also leads to a considerable increase in the number of IE protein-positive cells, suggesting that Sp100 likewise contributes to ND10-mediated viral repression (Adler, M., Tavalai, N., Stamminger, T., in preparation). Overall, these data support a model in which, comparable to HSV-1 infection, individual ND10 components function in counteracting the initiation of an efficient productive viral replication. This further underlines the notion of ND10 as mediators of an intrinsic immune defense against herpesvirus infections in general.

## 8. Gamma-Herpesviruses and ND10

### 8.1. Epstein-Barr Virus (EBV)

In common with all herpesviruses, EBV from the gamma-herpesvirus subfamily, possesses a biphasic life cycle consisting of a lytic and a latent phase. However, in contrast to HSV-1, its latent state is characterized by the expression of a set of proteins that are necessary for establishment and maintenance of latency [111]. Latent EBV infection, in addition, is strongly associated with several types of cancer, including nasopharyngeal carcinoma (NPC) [141]. While during latency, EBV genomes can be found in close association with interphase chromosomes, activation of the lytic replication cycle results in a spatial redistribution of viral DNA to ND10 [142]. Using in situ hybridization the authors also showed the development of replication centers starting at ND10 as replicating EBV genomes were frequently found beside this nuclear domain [142]. Subsequent studies identified the ori_lyt_ sequence as being required for the appearance of replicating episomes in association with PML-NBs [143]. However, the integrity of ND10 is no longer maintained as soon as productive EBV infection is initiated. The expression of lytic proteins is accompanied by a sequential redistribution of ND10 proteins starting with the dispersal of Sp100, hDaxx, and NDP55 which is then finally followed by PML relocalization [142–144]. Induction of productive EBV replication goes along with the expression of BZLF-1, the principal inducer of the lytic gene expression program [111]. Indeed, experiments carried out by Adamson and Kenney [144] illustrated, that BZLF-1 expression is sufficient to induce ND10 disruption by reducing the level of SUMOylated PML. BZLF-1, which is itself SUMO-1 modified, has been shown to compete with PML for limiting amounts of SUMO-1 [144]. In accordance with this, mutation of the SUMO-modification site of BZLF-1 abrogates desumoylation of PML [144]. However, this does not annihilate the capability of BZLF-1 to disperse ND10 after transient expression suggesting the existence of additional mechanisms for BZLF-1-mediated ND10 disruption. Further mutational analysis of BZLF-1 revealed a correlation between the capability of this protein to disperse Sp100 from ND10 and its efficiency to activate transcription via interaction with the ND10-located factor CBP [145].

Although initial studies suggested that EBV infection has no influence on ND10 during latency [142], recent evidence indicates that the latently expressed protein EBNA-LP (also designated as EBNA5) is likewise modifying the composition of ND10 accumulations. Already over ten years ago, EBNA-LP, which is important for EBV-mediated B-cell immortalization by functioning as a potent gene-specific coactivator of the viral transcriptional activator EBNA2 [146], was reported to colocalize with ND10 in EBV-positive lymphoblastoid cell lines [147]. Novel findings imply that EBNA-LP coactivates EBNA2 through binding of the ND10 factor Sp100, thereby selectively displacing it together with HP1α from PML-NBs [148]. Since both HP1α and Sp100 have been shown to possess transcriptional repressor activity [44,45,149], and expression of a mutant form of Sp100, that was unable to associate with ND10, resulted in coactivation of EBNA2 even in the absence of EBNA-LP [148], it has been speculated that the EBNA-LP-induced ND10 rearrangements may mitigate transcriptional barriers that prevent efficient expression of viral latent genes important for establishing latency. Furthermore, as IFN-αβtreatment of cells did not compromise EBNA-LP′s coactivator function it was suggested that EBNA-LP might play a role in EBV-evasion of IFN-mediated antiviral responses [150]. Interestingly, novel findings from Sivachandran *et al*. demonstrate that EBV latent infection in NPC cells is also associated with a modification of ND10 integrity and that EBNA1 is responsible for this effect by decreasing PML protein levels in a proteasome-dependent manner as well as through interaction with the cellular ubiquitin-specific protease USP7/HAUSP [151]. In addition, the data support a model in which EBNA1-mediated ND10 disruption promotes the survival of cells with DNA damage [151]. Thus, EBNA1 is considered as a viral factor that contributes to the establishment of NPC. In summary, these results are regarded as the first indication that modulation of ND10 might also be required for the establishment of nonproductive or latent herpesvirus infections as well as for the development of virus-induced cancers.

### 8.2. Kaposi’s Sarcoma-Associated Herpesvirus (KSHV)

Human herpesvirus 8 (HHV8), a gamma-herpesvirus also known as Kaposi′s sarcoma-associated herpesvirus (KSHV), is a lymphotropic virus involved in the pathogenesis of Kaposi′s sarcoma (KS), primary effusion lymphoma (PEL), and a subset of multicentric Castleman′s disease (MCDs). KSHV also encodes an early protein, K8, which is a distant evolutionary homologue of the EBV regulatory factor BZLF-1, that has been found to localize to ND10 and to establish replication compartments in association with this nuclear substructure [152,153]. However, unlike BZLF-1, K8 does not induce any changes to ND10 composition [153]. Consequently, since the genome replication of KSHV also occurs in close contact to PML-NBs, it seems to be a general feature of herpesviruses to initiate their replication in the near vicinity of ND10. Nevertheless, for a long time, KSHV was considered to be unique among herpesviruses in that it might not target ND10 for destruction. However, very recently, Marcos-Villa *et al*. identified the LANA2 protein of KSHV as a viral regulatory factor that is capable of inducing the disruption of ND10 [154]. As a mechanism for this, it was proposed that LANA2 mediates the proteasome-dependent degradation of PML by promoting SUMO2-based ubiquitylation of PML [154]. Moreover, the authors presented evidence that depletion of PML caused by LANA2 relieves PML-mediated transcriptional repression of cellular survivin, a host protein that directly contributes to the malignant progression of KSHV-infected PEL cells [154]. Taken together, these data strongly support the notion that, similar to EBV (see 8.1.), KSHV has developed strategies to neutralize ND10 function in order to promote cellular transformation and carcinogenesis.

### 8.3. Murine Gamma-Herpesvirus 68 (MHV-68)

Like KSHV, MHV-68 (also known as murid herpesvirus 4) belongs to the herpesvirus genus rhadinovirus. It is emerging as a suitable model to study basic biological questions of gamma-herpesvirus host interactions due to the fact that it naturally infects mice and establishes chronic infections in them.

Intriguingly, recent data suggested that the MHV-68 infection cycle is also negatively influenced by ND10 as viral replication results in the disassembly of this subnuclear structure [155,156]. The virion tegument protein ORF75c of MHV-68, a polypeptide with homology to the cellular formylglycinamide ribotide amidotransferase (FGARAT) enzyme, was identified as being responsible for this effect by inducing the proteasomal degradation of the main structural ND10-organizer PML [155]. Consequently, an ORF75c deletion mutant failed to destroy ND10 accumulations [156]. In accordance with the assumption that the ORF75c-induced depletion of PML is a means to annihilate the antiviral activity of ND10, MHV-68 replication turned out to be more robust in murine fibroblasts being deficient of PML [155]. However, although Ling and colleagues claimed that the degradation of PML by ORF75c is limited to murine fibroblasts and cannot be observed with human PML [155], we could also detect a complete elimination of the human PML variants upon infection of primary human fibroblast with MHV-68 (Tavalai, Full, Ensser and Stamminger, unpublished). Furthermore, the entire loss of PML as a consequence of MHV-68 infection presumably also accounted for a simultaneously observed disappearance of the low mobility isoforms of Sp100, comparable to the situation in HSV-1 infected cells (see 6.1.) or after depletion of the defining ND10 factor in PML-kd HFFs [14]. At the same time, MHV-68 also induced a significant reduction in hDaxx protein levels when compared to mock-infected cells. Although this effect on hDaxx is much less pronounced in comparison to MHV-68-mediated PML downregulation, it nevertheless mimics the findings for HCMV, where we were likewise never able to detect a total depletion of the transcriptional repressor hDaxx during the HCMV replicative cycle [129]. Thus, MHV-68 negatively affects the abundance of all three major ND10 constituents directly upon infection in order to efficiently circumvent the intrinsic antiviral response instituted by ND10. Given the fact, that MHV-68 naturally infects mice, it could represent a useful model system for investigation of ND10-herpesvirus interactions *in vivo*.

## 9. Concluding Remarks

ND10 have been identified as preferential replication sites of virtually all members of the herpesvirus family. However, the reason why herpesviruses interact with and commonly alter ND10 accumulations during their replicative cycle was for a long time quite controversially discussed. In principle, two conflicting theories had been established over the years that tried to explain the reason for the intimate virus-ND10 interaction during infection. Firstly, ND10 could harbor components or provide functions that are advantageous for the virus and support viral replication. Alternatively, ND10 structures could illustrate aggregations of proteins that compromise virus growth and which are therefore targeted by the virus for destruction to be inactivated.

In this review we summarized the rapidly growing body of literature that strongly supports the concept of ND10 as a potent antiviral defense mechanism of the cell that efficiently counteracts herpesvirus infections. Evidence continues to accumulate that, in principle, all three major ND10 components, PML, Sp100 as well as hDaxx constitute host factors with antiviral activities. This assumption is most clearly supported by work on herpesviruses like HSV-1 (see 6.1.) or HCMV (see 7.1.), which show an enhanced infectivity in the absence of either of these basic ND10 constituents. In addition, recent findings indicate that also factors which only transiently localize to PML-NBs, like the SWI/SNF protein ATRX, contribute to the antiviral response instituted by ND10. Thus, it is tempting to speculate that, besides the structure defining components, still more ND10-related factors exist with antiviral properties, which await to be identified, yet.

**Figure 1.**
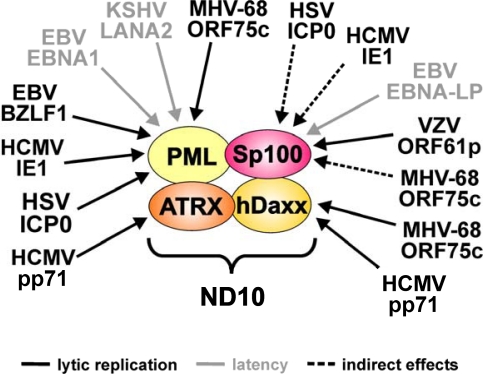
Targeting of the major ND10 proteins by herpesviral regulatory factors. The figure summarizes the herpesviral proteins that have been described to affect individual ND10 components. Arrows indicate which ND10 component is targeted by the respective factor. Grey lettering denotes viral proteins expressed during latency. Arrows with dashed lines indicate indirect effects of the respective factor.

Another characteristical aspect of cellular antiviral defense mechanisms is the fact that they are subject to viral countermeasures. Indeed, almost all herpesviruses for which replication has been linked to ND10, have evolved regulatory proteins that are capable of inactivating single ND10 components or disturbing the integrity of the whole subnuclear structure (see Figure 1 and Table 1). In many cases, these virus-induced alterations to ND10 composition have already been demonstrated to be important for the outcome of infection and correlate with the efficiency of viral propagation. Furthermore, it has been suggested that defeating ND10 function is not only a prerequisite for induction of an efficient productive replication but is also required for the establishment of latent infections as proposed in case of EBV (see 8.1.). Interestingly, recent data obtained from the herpesviruses EBV (see 8.1.) and KSHV (see 8.2.), in addition, imply that inactivation of ND10 may also contribute to the development of the tumors associated with these viruses. Consequently, ND10 seem to act at multiple steps of the virus life cycle in order to compromise the detrimental outcome of viral infections. Intriguingly, however, even closely related members of the herpesvirus family appear to have evolved their own specific strategies during virus-host co-evolution to cope with the antiviral aspects of ND10.

In summary, the future challenge will be to get a more detailed understanding of the underlying molecular mechanisms of ND10 as well as of the viral factors antagonizing its repressive function since elucidating the exact mode of action may help to develop novel therapeutic approaches against herpesviruses.

## Figures and Tables

**Table 1.** Overview of herpesviral regulatory proteins affecting individual ND10 components.

**Virus**	**Viral protein**	**ND10 target**	**Effects**
HSV-1	ICP0	PML	Proteasomal degradation of all isoforms
		Sp100	Loss of low mobility isoforms (indirect effect: linkage to PML metabolism)
VZV	ORF61p	Sp100	Downregulation of all isoforms
HCMV	IE1	PML	Loss of SUMOylated variants
		Sp100	Loss of low mobility isoforms (indirect effect: linkage to PML metabolism)
	pp71	hDaxx	Proteasomal degradation
		ATRX	Release from ND10
EBV	BZLF-1	PML	Loss of SUMOylated variants during lytic replication
	EBNA-LP	Sp100	Release from ND10
	EBNA1	PML	Proteasomal degradation during latent infection
KSHV	LANA2	PML	Proteasomal degradation during latent infection
MHV-68	ORF75c	PML	Proteasomal degradation of all isoforms
		Sp100	Loss of low mobility isoforms (indirect effect: linkage to PML metabolism)
		hDaxx	Downregulation
